# Power Indices Through Rotational Inertial Devices for Lower Extremity Profiling and Injury Risk Stratification in Professional Soccer Players: A Cross-Sectional Study

**DOI:** 10.3390/diagnostics15131691

**Published:** 2025-07-02

**Authors:** Álvaro Murillo-Ortiz, Javier Raya-González, Moisés Falces-Prieto, Samuel López-Mariscal, Francisco Javier Iglesias-García, Luis Manuel Martínez-Aranda

**Affiliations:** 1 Research Group on Sport and Physical Education for Personal and Social Development (GIDEPSO), Department of Specific Didactics, Faculty of Education Sciences and Psychology, University of Córdoba, 14071 Córdoba, Spain; alvaromurillo97@gmail.com; 2 GIR07, Research Group in Physical Activity and Sports Sciences, University Isabel I, 09003 Burgos, Spain; mfalpri@gmail.com; 3 High Performance Department KMSK Deinze, 9800 Deinze, Belgium; samuellopm@gmail.com (S.L.-M.); javi.iglesias30@gmail.com (F.J.I.-G.); 4 Department of Sports and Computer Sciences, Faculty of Sports Sciences, Universidad Pablo de Olavide, 41013 Seville, Spain; lmmarara@upo.es; 5 Research Group CTS563, Faculty of Education, Málaga University, 29071 Málaga, Spain; 6 Science-Based Training Research Group (SEJ-680), Physical Performance and Sports Research Center, Universidad Pablo de Olavide, 41013 Seville, Spain

**Keywords:** lower musculoskeletal strength, power indices, injury prevention, dominant limb, professional soccer players

## Abstract

**Background/Objectives**: Power indices may provide valuable information for performance and injury prevention in soccer players, so increasing the knowledge about them seems essential. Therefore, this study aimed to establish limb-specific normative values for flywheel-derived power indices in professional soccer players, while accounting for limb performance or ability, to explore the relationships between power indices across variables and to compare the power outcomes related to these indices between injured and non-injured players within four months post-assessment. **Methods**: Twenty-two male professional soccer players (age: 26.6 ± 4.6 years; competitive level: Belgian second division) were recruited from a single elite-tier club to participate in this cross-sectional diagnostic study. Participants underwent a standardized assessment protocol, executed in a rotational inertial device, comprising six unilateral exercises focused on the lower limbs: hip-dominant quadriceps (Qhip), knee-dominant quadriceps (Qknee), hip-dominant hamstrings (Hhip), knee-dominant hamstrings (Hknee), adductor (Add), and abductor (Abd). The testing session incorporated a randomized, counterbalanced design, with each exercise comprising two sets of eight maximal concentric–eccentric repetitions per limb. Leg dominance was operationally defined as the self-reported preferred limb for ball-striking tasks. Power indices were calculated from these exercises. **Results**: No significant differences in flywheel-derived power indices were found between limbs or between injured and non-injured players. However, significant correlations between indices were found in all power variables, with the Qhip:Qknee and Hhip:Hknee concentric ratios emerging as the most clinically actionable biomarkers for rapid screening. **Conclusions**: These results suggest the necessity of including more variables for injury prediction. Moreover, power indices could be considered based on the classification of limbs as “strong” or “weak”.

## 1. Introduction

In recent years, the study of lower-limb asymmetries in athletes has gained relevance in contemporary sports medicine research, particularly regarding their association with injury occurrence [1,2]. Despite this growing interest, emerging evidence suggests limited predictive validity of conventional asymmetry variables for stratifying injury risk [3], highlighting the need for novel biomarkers. Among the proposed alternatives, kinetic performance indices derived from multiplanar power profiles have emerged as a promising approach to assess sport-specific neuromuscular function [4,5].

The hamstring-to-quadriceps (H/Q) ratio, traditionally measured using isokinetic dynamometry [6], has been extensively studied in soccer populations [7]. However, this method fails to replicate the multiplanar, high-velocity demands of soccer-specific movements. These actions frequently involve rapid transitions between eccentric and concentric contractions (i.e., stretch-shortening cycles) during directional changes and ball striking [8]. As a result, there is growing agreement that sport-specific kinetic profiling requires assessment tools that better reflect functional movement patterns under ecologically valid loading conditions.

Rotational inertial devices offer superior ecological validity by quantifying concentric and eccentric power throughout the full range of motion [9,10]. These devices enable high-velocity, multiplanar movements that simulate soccer-specific tasks—key mechanisms behind non-contact injuries [11]. This is particularly relevant for eccentric lower-limb strength, which governs deceleration during sprinting and cutting maneuvers [12]. Conventional H/Q ratios, predominantly assessed via isokinetic dynamometry, focus on concentric strength and overlook eccentric control during high-risk tasks. Rotational inertial devices uniquely provide velocity-dependent resistance, requiring precise acceleration–deceleration modulation. This may offer greater sensitivity in detecting functional asymmetries than isokinetic methods [13]. Despite their increasing application in elite sports, standardized evaluation protocols and sport-specific normative data for flywheel metrics remain underdeveloped [14]. Given soccer players’ specific biomechanical demands, this population represents an ideal cohort for such research. However, power indices in professional soccer players remain largely unexplored, representing a critical gap in current knowledge [8].

Ongoing debates around injury risk prediction continue to highlight the limitations of conventional biomechanical metrics, particularly those based on isolated movement phases that neglect kinetic interdependencies across the musculoskeletal chain [8]. Although traditional H/Q ratio thresholds offer limited predictive value for hamstring injuries, their diagnostic capacity is further compromised by their inability to detect compensatory mechanisms across the kinetic chain, including the trunk, hips, ankles, and feet [6,15]. For example, athletes may present normal H/Q ratios yet remain vulnerable to ACL injuries due to insufficient adductor magnus activation during deceleration—allowing excessive femoral internal rotation [16].

Positional demands in soccer add further complexity. Wide midfielders often tolerate greater Q/Q asymmetry without increased injury risk—likely as a neuromuscular adaptation to repetitive crossing motions. In contrast, central defenders exceeding 12% asymmetry show a 4.2-fold increase in ACL injury risk due to frequent high-load pivoting under defensive pressure [17]. Compounding these challenges, isokinetic strength assessments have demonstrated poor discriminative validity in stratifying injury risk among soccer players [18], underscoring the need for integrative profiling frameworks that reflect sport-specific kinetic chains. Although rotational inertial devices show promise for assessing neuromuscular function under ecological conditions [14,19], prospective validation of their derived indices as predictive biomarkers remains necessary in elite soccer cohorts.

To address these gaps, this study employed a targeted monitoring protocol based on rotational inertial devices. Specifically, the study had three main aims: (1) to establish limb-specific normative values for inertial rotational devices’ power indices in professional soccer players, accounting for limb ability; (2) to map correlations between indices for each power variable; and (3) to compare power outcomes between injured and non-injured players within four months following assessment. Based on previous findings [18,19], we hypothesized that significant correlations would exist between exercise-specific power indices, and that these indices would differ between injured and non-injured players.

## 2. Materials and Methods

### 2.1. Study Design

A cross-sectional study design was conducted to (1) compare rotational power indices between dominant and non-dominant legs across multiple exercises performed on inertial devices, (2) analyze correlations among power indices derived from each exercise, and (3) evaluate differences in power indices between injured and non-injured professional soccer players. During a single assessment session, participants completed six exercises on a rotational inertial device in randomized order: quadriceps-dominant hip, hamstrings-dominant knee, hip abductor, quadriceps-dominant knee, hamstrings-dominant hip, and hip abductor exercises. Each exercise consisted of two sets of eight repetitions performed unilaterally. Leg dominance was defined as the preferred limb for ball striking, and the non-dominant leg as the supporting leg during kicking [20]. Testing was conducted at the club’s training facilities (17–22 °C; 60–70% relative humidity) between 9:00 and 11:00 a.m. under the supervision of the strength and conditioning staff. A standardized warm-up protocol preceded testing, comprising cycling, joint mobility drills, dynamic stretching, bilateral and unilateral jumps, and a submaximal familiarization set on the inertial device. Participants were instructed to maintain habitual dietary practices, ensure proper hydration and carbohydrate intake in the 24 h prior to testing, and avoid caffeinated beverages on the day of the session. All lower-limb non-contact injuries occurring within four months after the assessment were recorded by the club’s medical staff in accordance with the International Olympic Committee consensus statement for soccer [21]. Information about number of absence days, severity, type (in terms of damaged tissue), location, and whether the injury occurred during training or match-play were registered.

### 2.2. Participants

Twenty-two male professional Belgian soccer players (age = 26.6 ± 4.6 years; height: 182.1 ± 6.1 cm; weight: 75.6 ± 6.3 kg; BMI = 22.8 ± 1.1 kg/m^2^; fat mass = 9.1 ± 0.6%; fat-free mass = 68.4 ± 5.6 kg), all members of the same team, participated in this study. During the intervention period, the team competed in the Belgian second division (Challenger Pro League). An a priori power analysis was conducted using G*Power (version 3.1.9.2, Universität Kiel, Kiel, Germany), indicating that a minimum of 20 participants was required to detect a large effect (ES = 0.90) with 84% power (1 − β = 0.84) at an alpha level of 0.05. Inclusion criteria were: (1) complete all assessment exercises (two sets per movement), (2) absence of acute or chronic injuries (e.g., muscle-tendon or joint-related), and (3) at least five years of licensed soccer experience. Prior to participation, players received detailed information about the study objectives, procedures, and risks, and provided written informed consent. The protocol was approved by the Research Ethics Committee of the University of Córdoba (Code: CEIH-24-50) and conformed to the Declaration of Helsinki.

### 2.3. Procedures

**Figure 1:**
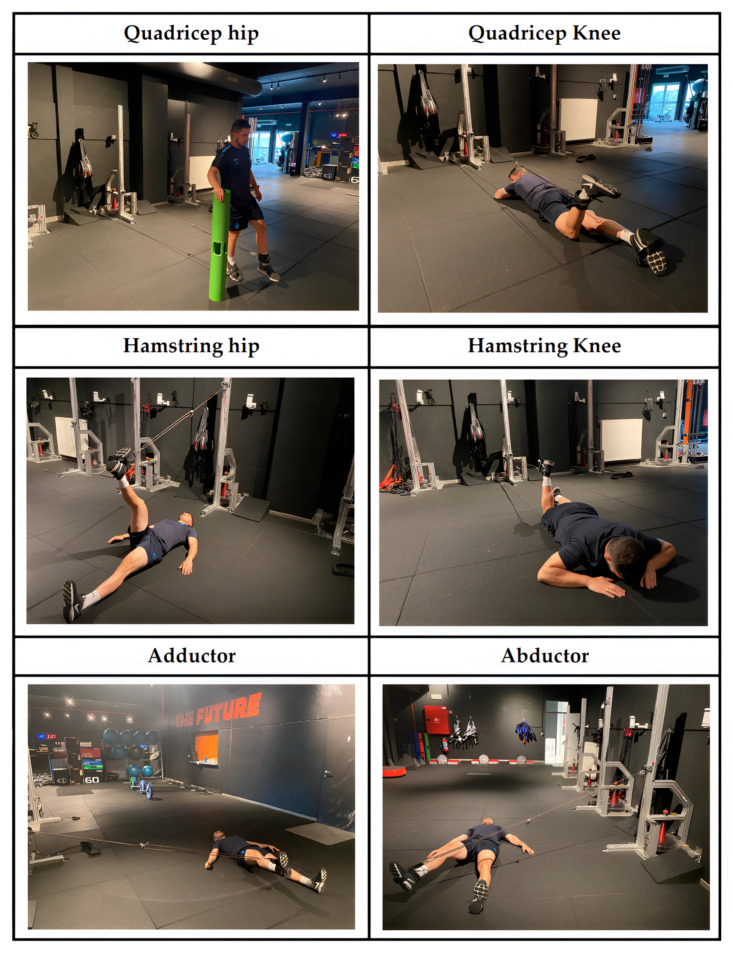
Exercises by muscle group.

A single testing session was conducted to quantify kinetic and kinematic outputs during unilateral lower-limb resistance exercises performed on an inertial flywheel system. The protocol comprised six distinct movements targeting the quadriceps (hip extension and knee extension), hamstrings (hip flexion and knee flexion), hip adductors, and hip abductors (Figure 1). Participants performed two randomized sets of eight repetitions per exercise (inertial load: 0.0335 kg·m^2^), preceded by two additional warm-up repetitions to accommodate cone acceleration [20]. Four-minute rest intervals separated sets. Athletes were instructed to maximize concentric velocity and delay braking until the final third of the eccentric phase [22]. Exercises were executed using the Pulley Pro C3 inertial trainer (Proinertial^®^, Barcelona, Spain), equipped with a rotary encoder (Chronojump, Barcelona, Spain) interfaced with proprietary software (v2.3.0-2423) for data acquisition [23,24]. Kinetic variables, including mean and peak power, velocity, and force, were sampled across concentric (CON) and eccentric (ECC) phases. According to Mak et al. [25], flywheel testing is reliable (ICC = 0.66–0.99, r = 0.69–0.97, α = 0.85–0.98) and valid for the athletic population when subjects undergo two familiarization sessions. For analytical purposes, the repetition with the highest mean concentric power from the second set was selected. Testing was conducted under standardized conditions, with real-time biofeedback via mirrors and technical oversight by certified coaches [20]. Based on the included exercises, the following indices were calculated: quadriceps hip/quadriceps knee (Qhip:Qknee); hamstring hip/hamstring knee (Hhip:Hknee); quadricep hip/hamstring hip (Qhip:Hhip); quadricep knee/hamstring knee (Qknee:Hknee); adductor/abductor (Add:Abd).

### 2.4. Statistical Analysis

Data are presented as mean ± standard deviations (SD). Normality and homogeneity of variances were verified using Shapiro–Wilk and Levene’s tests. Paired-sample *t*-tests were used to compare power indices between dominant and non-dominant legs, and between injured and non-injured players. Effect sizes (ES) were calculated using Cohen’s ES to assess the magnitude of the effects. ES were interpreted as follows: <0.2, trivial; 0.20 to 0.49, small; 0.50 to 0.80, moderate; and >0.80, large [26]. Pearson’s correlation coefficients (*r*), with a 90% confidence interval, were used to examine associations between power outputs across exercises, and interpreted as: <0.1 = trivial, 0.1–0.3 = small, 0.3–0.5 = moderate, 0.5–0.7 = large, 0.7–0.9 = very large, and >0.9 = nearly perfect [27]. Statistical analyses were conducted using JASP (v0.18.1; JASP Team, Amsterdam, The Netherlands), with significance set at *p* < 0.05. A Bonferroni correction was applied for multiple comparisons, adjusting the significance threshold to *p* < 0.012.

## 3. Results

### 3.1. Between Limbs’ Differences

Table 1, Table 2 and Table 3 present the comparison between dominant and non-dominant limbs with regard to power indices. No significant differences were observed in any comparison.

### 3.2. Correlations Between Power Indices

**Figure 2:**
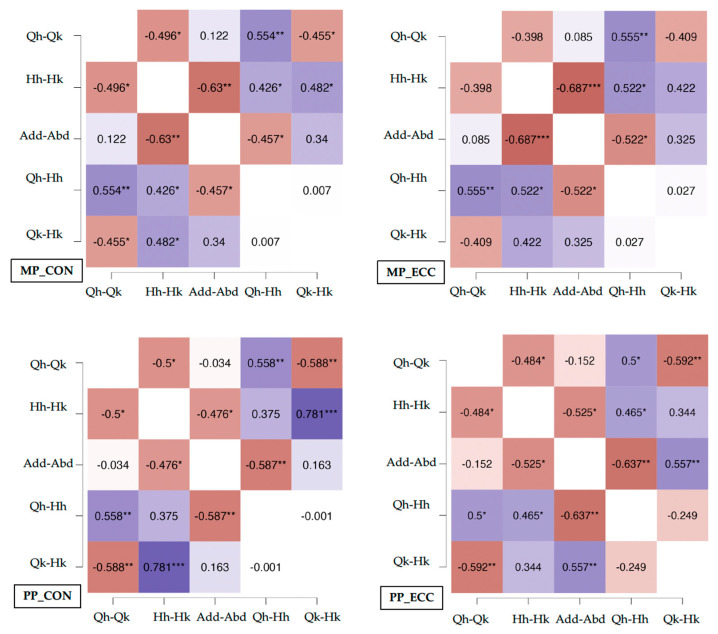
Correlations between indices for each power variable. MP_CON = mean power concentric; MP_ECC = mean power eccentric; PP_CON = peak power concentric; PP_ECC = peak power eccentric. The colors indicate the strength of the correlation, with darker shades representing stronger relationships. Purple tones indicate positive correlations; orange tones indicate negative correlations. *: significance level at *p* < 0.05; **: significance level at *p* < 0.01; ***: significance level at *p* < 0.001.

Correlations between power indices across variables are presented in Figure 2. For concentric mean power, significant relationships (*p* < 0.05) were identified between Qhip:Qknee and Hhip:Hknee, Qhip:Hhip and Qknee:Hknee; between Hhip:Hknee and Qhip:Hhip, Qknee:Hknee and Add:Abd; and between Add:Abd and Qhip:Hhip. Similarly, eccentric mean power exhibited significant correlations (*p* < 0.05) between Qhip:Qknee and Qhip:Hhip; between Hhip:Hknee and Qhip:Hhip, Qknee:Hknee and Add:Abd; and between Add:Abd and Qhip:Hhip.

Concentric peak power demonstrated analogous patterns, with significant associations (*p* < 0.05) observed between Qhip:Qknee and Hhip:Hknee, Qhip:Hhip and Qknee:Hknee; between Hhip:Hknee and Qknee:Hknee and Add:Abd; and between Add:Abd and Qhip:Hhip. Finally, eccentric peak power revealed significant correlations (*p* < 0.05) between Qhip:Qknee and Hhip:Hknee, Qhip:Hhip and Qknee:Hknee; between Hhip:Hknee and Qhip:Hhip and Add:Abd; and between Add:Abd and Qhip:Hhip and Qknee:Hknee.

### 3.3. Differences Between Injured and Non-Injured Players

Table 4, Table 5 and Table 6 show the comparison between injured and non-injured players with regard to power indices. No significant differences were observed in any comparison.

## 4. Discussion

The aim of this study was three-fold: (1) to establish limb-specific normative values for flywheel-derived power indices in professional soccer players, accounting for limb ability; (2) to map the intercorrelations between indices for each power variable; and (3) to compare the power outcomes related to these indices between injured and non-injured players within four months post-assessment. As a novel contribution, the study compared concentric and eccentric power indices across hip, knee, and stabilizer muscle movements. The main results revealed the following: firstly, there was no significant difference in any index between dominant and non-dominant limbs; secondly, there were moderate to large correlations between some indices across exercises; and finally, there were no differences in any power index between injured and non-injured players during the follow-up period.

### 4.1. Between Limbs’ Differences

The analysis of inter-limb power differences in soccer has diagnostic relevance due to the sport’s inherent biomechanical demands, which impose divergent functional roles on the lower extremities [28]. The dominant leg predominantly executes high-velocity, precision-dependent actions such as ball striking and curved passing, while the non-dominant leg assumes a stabilizing role during weight-bearing tasks, including deceleration, pivoting, and dynamic postural control [29]. This task-specific specialization could, in theory, lead to distinct neuromuscular adaptations, expressed as clinically relevant asymmetries in strength, in the rate of force development, or in eccentric-to-concentric force ratios [30].

However, our results showed no statistically significant differences (*p* > 0.05) in concentric or eccentric power indices between limbs in any of the tested exercises. This aligns with recent evidence suggesting that functional limb dominance does not necessarily result in mechanical asymmetry in elite soccer players. One possible explanation lies in the sport’s training context: both legs are regularly exposed to high-intensity, multidirectional loads during drills (e.g., repeated sprints, cutting maneuvers) and matches, which may promote compensatory adaptations that equalize neuromuscular performance [31]. For example, the stabilizing role of the non-dominant leg during kicking may require eccentric hamstring strength similar to the concentric quadriceps strength of the dominant leg, ultimately balancing mechanical output between limbs [32]. These findings provide practical implications for performance testing in soccer players. Given the absence of significant differences, they suggest that performance evaluations might be conducted using only one leg. Nonetheless, future studies should classify limbs as “strong” or “weak” to draw more definitive conclusions.

### 4.2. Correlations Between Power Indices

The development of diagnostically robust assessment protocols in soccer is essential to understand how power indices relate across biomechanical variables and exercise modalities, especially within time-efficient, athlete-centered testing frameworks [33]. Our findings reveal significant correlations between key power indices, most notably in concentric-phase quadriceps-to-hamstring ratios at the hip and knee joints (e.g., Qhip:Qknee and Hhip:Hknee ratios). These strong associations likely reflect the functional interdependence of these muscle groups during sport-specific movements such as sprint acceleration and deceleration, where coordinated hip–knee extension and flexion govern both force production (via concentric quadriceps action) and force absorption (via eccentric hamstring braking) [34].

In contrast, weaker correlations were observed in eccentric indices and in stabilizer muscle groups (e.g., adductor/abductor ratios). This may be due to the complexity of eccentric motor control, which involves greater proprioceptive input and more variable motor unit recruitment strategies [35]. Additionally, adductor–abductor metrics may show higher variability between sessions because these muscles primarily respond to destabilizing forces rather than being involved in maximal force production, making them less suited to standardized assessments [36]. From a practical diagnostic standpoint, the Qhip:Qknee and Hhip:Hknee concentric ratios appear to be clinically useful biomarkers for rapid screening. These indices condense complex neuromuscular information into simple metrics, allowing practitioners to assess overall mechanical output (e.g., power decline during repeated sprints) or detect compensatory strategies (e.g., reliance on knee-dominant mechanics) without the need for exhaustive testing protocols.

### 4.3. Differences Between Injured and Non-Injured Players

Identifying biomechanical precursors to sports injuries requires comparing power indices between injured and non-injured athletes, as these markers may reveal subclinical deficits in neuromuscular control or load tolerance [37]. In this study, however, no statistically significant differences (*p* > 0.05) were detected between groups across any mechanical index derived from flywheel testing, including eccentric/concentric ratios, impulse, or rate of force development. This null finding underscores the multifactorial etiology of sports injuries, wherein biomechanical factors interact dynamically with extrinsic loads (e.g., training volume, competition density), intrinsic vulnerabilities (e.g., unresolved prior injuries, collagen remodeling status), and psychosocial stressors (e.g., competitive anxiety, sleep quality) [38].

Although rotational inertial devices provide high ecological validity due to their sport-specific resistance profiles and capacity to induce eccentric overload [8], their predictive utility for injury remains limited due to methodological constraints. The eccentric phase, despite its clinical relevance to injury mechanisms (e.g., hamstring strains during terminal swing), exhibits high intra-subject variability, likely attributable to inconsistencies in braking mechanics, proprioceptive engagement, or motor unit synchronization during rapid deceleration [39]. Such variability can mask subtle group differences in eccentric capacity, which may only become evident under fatigue or during unanticipated movements not captured in controlled testing conditions.

These findings support a shift toward integrative diagnostic models that combine flywheel-derived data with additional biomarkers, such as tissue-specific imaging (e.g., ultrasound of muscle architecture), biochemical stress indicators (e.g., cortisol, creatine kinase), and cognitive–motor tests (e.g., reactive balance assessments). Future studies should explore longitudinal evaluations using rotational inertial devices and develop protocols that simulate chaotic mechanical demands, such as unexpected inertial load changes or reactive directional shifts.

Despite certain methodological limitations, the results of the present study provide a valuable foundation for improving the understanding of the diagnostic potential of power indices obtained using flywheel devices in professional soccer players. First, a single team was involved in the study, which could have influenced the results, mainly related to correlations, due to the sample size. However, it is difficult to obtain larger samples for similar studies with professional soccer players. Second, no force-plates were used, which could have provided relevant information during inertial device assessments [40]. Third, the experimental design employed a single assessment session, precluding longitudinal analysis of chronic neuromuscular adaptations or transient performance fluctuations associated with cumulative fatigue. Finally, the internal and external load during the follow-up period were not analyzed, which could impact injury occurrence [41]. Advancing the diagnostic potential of inertial-based assessments will require longitudinal designs with broader, more diverse cohorts, as well as the inclusion of contextual factors like prior injury history, positional demands, and accumulated match exposure.

## 5. Conclusions

The findings revealed no significant differences in limb-specific or injury-stratified power indices, which suggests the need to find novel approaches related to injury prediction. Instead, the strong intra-limb correlations observed in this study underscore the need to redefine risk assessment frameworks toward metrics of intra-limb kinetic synergy. Future research should prioritize longitudinal designs to determine whether intra-limb synergy thresholds improve injury prognostication in elite soccer populations, especially when evaluated in the context of sport-specific movement patterns.

## Figures and Tables

**Table 1:** Descriptive values and comparison for quadricep and hamstring indexes.

Variable	Index					
	Qhip:Qknee			Hhip:Hknee		
	Dominant	Non-Dominant	Pairwise Comparisons (ES; *p*)	Dominant	Non-Dominant	Pairwise Comparisons (ES; *p*)
Mean power CON	1.55 ± 0.50	1.56 ± 0.45	0.028; 0.897	0.81 ± 0.21	0.839 ± 0.16	0.149; 0.493
Mean power ECC	1.55 ± 0.47	1.58 ± 0.46	0.069; 0.749	0.77 ± 0.23	0.79 ± 0.20	0.094; 0.665
Peak power CON	1.43 ± 0.45	1.39 ± 0.32	0.099; 0.646	0.84 ± 0.27	0.87 ± 0.18	0.121; 0.576
Peak power ECC	1.20 ± 0.44	1.25 ± 0.49	0.130; 0.549	1.11 ± 0.39	1.15 ± 0.46	0.078; 0.718

Abbreviations: Qhip = hip dominant quadriceps exercise; Qknee = knee dominant quadriceps exercise; Hhip = hip dominant hamstring exercise; Hknee = knee dominant hamstring exercise; ES = effect size.

**Table 2:** Descriptive values and comparison for hip and knee indexes.

Variable	Index					
	Qhip:Hhip			Qknee:Hknee		
	Dominant	Non-Dominant	Pairwise Comparisons (ES; *p*)	Dominant	Non-Dominant	Pairwise Comparisons (ES; *p*)
Mean power CON	1.21 ± 0.34	1.27 ± 0.34	0.261; 0.235	1.87 ± 0.52	1.87 ± 0.36	0.090; 0.997
Mean power ECC	1.17 ± 0.39	1.22 ± 0.38	0.206; 0.344	1.81 ± 0.38	1.79 ± 0.43	0.046; 0.831
Peak power CON	1.15 ± 0.31	1.20 ± 0.37	0.156; 0.472	1.77 ± 0.31	1.75 ± 0.29	0.243; 0.266
Peak power ECC	1.25 ± 0.44	1.34 ± 0.59	0.192; 0.379	1.63 ± 0.61	1.48 ± 0.71	0.195; 0.371

Abbreviations: Qhip = hip dominant quadriceps exercise; Qknee = knee dominant quadriceps exercise; Hhip = hip dominant hamstring exercise; Hknee = knee dominant hamstring exercise; ES = effect size.

**Table 3:** Descriptive values and comparison for adductor/abductor index.

Variable	Index		
	Add:Abd		
	Dominant	Non-Dominant	Pairwise Comparisons (ES; *p*)
Mean power CON	2.45 ± 0.66	2.31 ± 0.64	0.412; 0.067
Mean power ECC	2.47 ± 0.74	2.35 ± 0.69	0.379; 0.090
Peak power CON	2.29 ± 0.49	2.08 ± 0.50	0.422; 0.061
Peak power ECC	1.59 ± 0.72	1.40 ± 0.76	0.291; 0.187

Abbreviations: Add = adductor exercise; Abd = abductor exercise; ES = effect size.

**Table 4:** Descriptive values and comparison for quadriceps and hamstring indexes between non-injured and injured players.

Variable	Index					
	Qhip:Qknee			Hhip:Hknee		
	Non-Injured Players	Injured Players	Pairwise Comparisons (ES; *p*)	Non-Injured Players	Injured Players	Pairwise Comparisons (ES; *p*)
Mean power CON	1.43 ± 0.30	1.66 ± 0.63	0.480; 0.274	0.79 ± 0.21	0.83 ± 0.22	0.168; 0.697
Mean power ECC	1.46 ± 0.37	1.64 ± 0.56	0.381; 0.383	0.75 ± 0.22	0.80 ± 0.24	0.216; 0.618
Peak power CON	1.26 ± 0.27	1.60 ± 0.53	0.812; 0.071	0.88 ± 0.32	0.80 ± 0.21	0.278; 0.522
Peak power ECC	1.19 ± 0.33	1.20 ± 0.55	0.028; 0.949	0.98 ± 0.33	1.24 ± 0.42	0.692; 0.121

Abbreviations: Qhip = hip dominant quadriceps exercise; Qknee = knee dominant quadriceps exercise; Hhip = hip dominant hamstring exercise; Hknee = knee dominant hamstring exercise; ES = effect size.

**Table 5:** Descriptive values and comparison for hip and knee indexes between non-injured and injured players.

Variable	Index					
	Qhip:Hhip			Qknee:Hknee		
	Non-Injured Players	Injured Players	Pairwise Comparisons (ES; *p*)	Non-Injured Players	Injured Players	Pairwise Comparisons (ES; *p*)
Mean power CON	1.10 ± 0.28	1.31 ± 0.37	0.635; 0.152	1.95 ± 0.39	1.85 ± 0.44	0.240; 0.580
Mean power ECC	1.07 ± 0.35	1.27 ± 0.42	0.501; 0.254	1.86 ± 0.31	1.76 ± 0.45	0.248; 0.568
Peak power CON	1.06 ± 0.22	1.24 ± 0.36	0.608; 0.169	2.03 ± 0.62	1.71 ± 0.36	0.641; 0.148
Peak power ECC	1.12 ± 0.36	1.38 ± 0.50	0.582; 0.187	1.64 ± 0.46	1.61 ± 0.76	0.043; 0.921

Abbreviations: Qhip = hip dominant quadriceps exercise; Qknee = knee dominant quadriceps exercise; Hhip = hip dominant hamstring exercise; Hknee = knee dominant hamstring exercise; ES = effect size.

**Table 6:** Descriptive values and comparison for adductor/abductor index between non-injured and injured players.

Variable	Index		
	Add:Abd		
	Non-Injured Players	Injured Players	Pairwise Comparisons (ES; *p*)
Mean power CON	2.63 ± 0.84	2.27 ± 0.37	0.548; 0.214
Mean power ECC	2.68 ± 0.91	2.25 ± 0.45	0.596; 0.178
Peak power CON	2.40 ± 0.56	2.18 ± 0.41	0.441; 0.314
Peak power ECC	1.86 ± 0.84	1.32 ± 0.50	0.784; 0.081

Abbreviations: Add = adductor exercise; Abd = abductor exercise; ES = effect size.

## Data Availability

The data are available upon request to the corresponding author.

## Abbreviations

The following abbreviations are used in this manuscript:

Qhip	Quadriceps hip
Hhip	Hamstring hip
Qknee	Quadriceps knee
Hknee	Hamstring knee
ABD	Abduction
ADD	Adduction
CON	Concentric
ECC	Eccentric

## References

[1] Carvalho A., Brown S., Abade E. (2016). Evaluating Injury Risk in First and Second League Professional Portuguese Soccer: Muscular Strength and Asymmetry. J. Hum. Kinet..

[2] Mokha M., Sprague P.A., Gatens D.R. (2016). Predicting Musculoskeletal Injury in National Collegiate Athletic Association Division II Athletes from Asymmetries and Individual-Test versus Composite Functional Movement Screen Scores. J. Athl. Train..

[3] Helme M., Tee J., Emmonds S., Low C. (2021). Does Lower-Limb Asymmetry Increase Injury Risk in Sport? A Systematic Review. Phys. Ther. Sport.

[4] Dauty M., Menu P., Fouasson-Chailloux A., Ferréol S., Dubois C. (2016). Prediction of Hamstring Injury in Professional Soccer Players by Isokinetic Measurements. Muscles Ligaments Tendons J..

[5] Dhahbi W., Ben Saad H., Dergaa I., Souaifi M., Chamari K. (2024). Injury Profiling in Male Police Cadets During Initial Training Phase: A Retrospective Cohort Study. Am. J. Men’s Health.

[6] Kellis E., Sahinis C., Baltzopoulos V. (2023). Is Hamstrings-to-Quadriceps Torque Ratio Useful for Predicting Anterior Cruciate Ligament and Hamstring Injuries? A Systematic and Critical Review. J. Sport Health Sci..

[7] O’Donnell S.R., Eitan D.N., Roper J.L. (2020). A Comparison of Quadriceps-to-Hamstrings Ratios during Isokinetic Testing, Cutting, and Drop Landings in Male Soccer Players. Int. J. Exerc. Sci..

[8] Raya-González J., Castillo D., Beato M. (2021). The Flywheel Paradigm in Team Sports. Strength Cond. J..

[9] Berg H.E., Tesch A. (1994). A Gravity-Independent Ergometer to Be Used for Resistance Training in Space. Aviat. Space Environ. Med..

[10] Norrbrand L., Tous-Fajardo J., Vargas R., Tesch P.A. (2011). Quadriceps Muscle Use in the Flywheel and Barbell Squat. Aviat. Space Environ. Med..

[11] Malone S., Owen A., Mendes B., Hughes B., Collins K., Gabbett T.J. (2018). High-Speed Running and Sprinting as an Injury Risk Factor in Soccer: Can Well-Developed Physical Qualities Reduce the Risk?. J. Sci. Med. Sport.

[12] van Dyk N., Bahr R., Burnett A.F., Whiteley R., Bakken A., Mosler A., Farooq A., Witvrouw E. (2017). A Comprehensive Strength Testing Protocol Offers No Clinical Value in Predicting Risk of Hamstring Injury: A Prospective Cohort Study of 413 Professional Football Players. Br. J. Sports Med..

[13] Delaney J.A., Cummins C.J., Thornton H.R., Duthie G.M. (2017). Importance, Reliability and Usefulness of Acceleration Measures in Team Sports. J. Strength Cond. Res..

[14] Beato M., Fleming A., Coates A., Dello Iacono A. (2021). Validity and Reliability of a Flywheel Squat Test in Sport. J. Sports Sci..

[15] Dhahbi W., Materne O., Chamari K. (2025). Rethinking Knee Injury Prevention Strategies: Joint-by-Joint Training Approach Paradigm versus Traditional Focused Knee Strengthening. Biol. Sport.

[16] Izovska J., Hank M., Cabell L., Kalata M., Bujnovsky D., Zahalka F., Maly T. (2022). The Hamstring and ACL Injury Incidence during a Season Is Not Directly Related to Preseason Knee Strength Ratios in Elite Male Soccer Players. Appl. Sci..

[17] Ruas C.V., Brown L.E., Pinto R.S. (2015). Lower-Extremity Side-to-Side Strength Asymmetry of Professional Soccer Players According to Playing Position. Kinesiology.

[18] Martins F., França C., Sarmento H., Henriques R., Przednowek K., Nascimento M.D.M., Marques A., Ihle A., Gouveia É.R. (2024). Lower Limbs Strength Variations between Injured and Non-Injured Professional Soccer Players. Sci. Prog..

[19] McErlain-Naylor S.A., Beato M. (2020). Concentric and Eccentric Inertia–Velocity and Inertia–Power Relationships in the Flywheel Squat. J. Sports Sci..

[20] Raya-González J., Castillo D., Domínguez-Díez M., Hernández-Davó J.L. (2020). Eccentric-Overload Production during the Flywheel Squat Exercise in Young Soccer Players: Implications for Injury Prevention. Int. J. Environ. Res. Public Health.

[21] Waldén M., Mountjoy M., McCall A., Serner A., Massey A., Tol J.L., Bahr R., D’Hooghe M., Bittencourt N., Della Villa F. (2023). Football-Specific Extension of the IOC Consensus Statement: Methods for Recording and Reporting of Epidemiological Data on Injury and Illness in Sport 2020. Br. J. Sports Med..

[22] Sabido R., Hernández-Davó J.L., Botella J., Navarro A., Tous-Fajardo J. (2017). Effects of Adding a Weekly Eccentric-Overload Training Session on Strength and Athletic Performance in Team-Handball Players. Eur. J. Sport Sci..

[23] Illera-Domínguez V., Nuell S., Carmona G., Padullés J.M., Padullés X., Lloret M., Cussó R., Alomar X., Cadefau J.A. (2018). Early Functional and Morphological Muscle Adaptations during Short-Term Inertial-Squat Training. Front. Physiol..

[24] Mariscal S.L., Gómez Á.R., Becerra M.O., Arrones L.S. (2024). Análisis y Relación Entre La Composición Corporal y Variables de Rendimiento En Jugadoras de Fútbol Sala. Rev. Iberoam. Cienc. Act. Física Deporte.

[25] Mak M., Bishop C., Beato M. (2025). Validity and Reliability of Flywheel Resistance Technology as an Assessment Method and Its Association with Sports Performance and Asymmetry: A Systematic Review. J. Strength Cond. Res..

[26] Cohen J. (1988). Statistical Power Analysis for the Behavioural Sciences.

[27] Hopkins W.G. (2007). A Spreadsheet for Deriving a Confidence Interval, Mechanistic Inference and Clinical Inference from a p Value. Sportscience.

[28] Raya-González J., Bishop C., Gómez-Piqueras P., Veiga S., Viejo-Romero D., Navandar A. (2020). Strength, Jumping, and Change of Direction Speed Asymmetries Are Not Associated With Athletic Performance in Elite Academy Soccer Players. Front. Psychol..

[29] Haddad M., Abbes Z., Zarrouk N., Aganovic Z., Hulweh A., Moussa-Chamari I., Behm D.G. (2023). Difference Asymmetry between Preferred Dominant and Non-Dominant Legs in Muscular Power and Balance among Sub-Elite Soccer Players in Qatar. Symmetry.

[30] Theodorou E., Tryfonidis M., Zaras N., Hadjicharalambous M. (2023). Musculoskeletal Asymmetries in Young Soccer Players: 8 Weeks of an Applied Individual Corrective Exercise Intervention Program. Appl. Sci..

[31] Jukic I., Calleja-González J., Cuzzolin F., Sampaio J., Cos F., Milanovic L., Krakan I., Ostojic S., Olmo J., Requena B. (2021). The 360° Performance System in Team Sports: Is It Time to Design a “Personalized Jacket” for Team Sports Players?. Sports.

[32] Cerrah A.O., Şimsek D., Soylu A.R., Nunome H., Ertan H. (2024). Developmental Differences of Kinematic and Muscular Activation Patterns in Instep Soccer Kick. Sports Biomech..

[33] Asimakidis N.D., Mukandi I.N., Beato M., Bishop C., Turner A.N. (2024). Assessment of Strength and Power Capacities in Elite Male Soccer: A Systematic Review of Test Protocols Used in Practice and Research. Sports Med..

[34] Lees A., Nolan L. (1998). The Biomechanics of Soccer: A Review. J. Sports Sci..

[35] Bittmann F.N., Dech S., Schaefer L.V. (2023). Another Way to Confuse Motor Control: Manual Technique Supposed to Shorten Muscle Spindles Reduces the Muscular Holding Stability in the Sense of Adaptive Force in Male Soccer Players. Brain Sci..

[36] Markovic G., Šarabon N., Pausic J., Hadžić V. (2020). Adductor Muscles Strength and Strength Asymmetry as Risk Factors for Groin Injuries among Professional Soccer Players: A Prospective Study. Int. J. Environ. Res. Public Health.

[37] Ruiz-Pérez I., Raya-González J., López-Valenciano A., Robles-Palazón F.J., Ayala F. (2023). Physical Differences between Injured and Non-Injured Elite Male and Female Futsal Players. Appl. Sci..

[38] Hägglund M., Waldén M., Ekstrand J. (2013). Risk Factors for Lower Extremity Muscle Injury in Professional Soccer. Am. J. Sports Med..

[39] Fernández-Valdés B., Sampaio J., Exel J., González J., Tous-Fajardo J., Jones B., Moras G. (2020). The Influence of Functional Flywheel Resistance Training on Movement Variability and Movement Velocity in Elite Rugby Players. Front. Psychol..

[40] Dhahbi W., Chaouachi A., Cochrane J., Chèze L., Chamari K. (2017). Methodological Issues Associated with the Use of Force Plates When Assessing Push-Ups Power. J. Strength Cond. Res..

[41] Dhahbi W., Chaabene H., Pyne D.B., Chamari K. (2024). Standardizing the Quantification of External Load Across Different Training Modalities: A Critical Need in Sport-Science Research. Int. J. Sports Physiol. Perform..

